# Supplementary material for: Understanding the unique S-scheme charge migration in triazine/heptazine crystalline carbon nitride homojunction
Source: Nat Commun. 2023 Jul 3;14:3901. doi: 10.1038/s41467-023-39578-z (PMC10317968; doi:10.1038/s41467-023-39578-z)
Supplement: Supplementary file 1 — Supplementary Information [file 41467_2023_39578_MOESM1_ESM.pdf]

Understanding the unique S-scheme charge migration in triazine/heptazine crystalline carbon nitride homojunction

Fang Li, [1] *Xiaoyang Yue,* [1] *Yulong Liao,* [1] *Liang Qiao,* [2*] *Kangle Lv,* [3*] *& Quanjun Xiang* [1]*

1 State Key Laboratory of Electronic Thin Film and Integrated Devices, School of Electronic Science and Engineering, University of Electronic Science and Technology of China, Chengdu 610054, P. R. China

E-mail: xiangqj@uestc.edu.cn;

2 School of Physics, University of Electronic Science and Technology of China, Chengdu 610054, PR China

E-mail: Liang.qiao@uestc.edu.cn;

3 Key Laboratory of Resources Conversion and Pollution Control of the State Ethnic Affairs Commission, College of Resources and Environment, South-Central Minzu University, Wuhan 430074, China

E-mail: lvkangle@mail.scuec.edu.cn;

Table of contents

2. Supplementary Table. 1 Relationship between vacuum layer thickness and work function of the sample.

3. Supplementary Table. 1 Energy band structure of crystalline carbon nitride. Band position of the sample.

4. Supplementary Table. 2 Composition and ratio of crystal phase in crystallized carbon nitride homojunction. The relationship between the ratio of triazine and heptazine phase and the peak area.

5. Supplementary Table. 3 Detailed information about the structural model. Types and number of atoms in the structural model

6. Supplementary Table. 3 Evaluation of photocatalytic activity. Assessment of CO_2 photoconversion activity over prepared samples.

7. Supplementary Fig. 1 Morphological characterization of the prepared sample. **a** SEM images of TCN, **b** HCN, and **c** TH1:4.

8. Supplementary Fig. 2 Simulated STM images. **a** Simulated STM images for TCN and **b** HCN along with the corresponding crystal structures.

9. Supplementary Fig. 3 Electrostatic self-Assembly synthesis of TH1:4. **a** Zeta potential of the as-prepared samples. **b** Illustration of the synthesis process of TH1:4. After hydrochloric acid treatment, the positively charged HCl-HCN and the negatively charged TCN were assembled by electrostatic self-assembly into homojunction.

10. Supplementary Fig. 4 Characterization of elemental content of HCN samples before and after acid treatment. Comparison of elemental content in HCN samples (before acid washing) and HHCN (after acid washing) samples.

11. Supplementary Fig. 5 Element distribution image of TH1:4. a The total element distribution image of TH1:4. Elemental mapping images for b C, c N, d O, e Cl, and f K.

12. Supplementary Fig. 6 STEM and HRTEM characterization ofTH1:4. a STEM images of TH1:4. **b, c and d** HRTEM images of TH1:4. **e** Schematic diagram of atomic arrangement at interface.

13. Supplementary Fig. 7 Differences in work functions due to different thicknesses of vacuum layers. a Work function of (002) crystal plane for HCN and b (002) crystal plane for TCN.

14. Supplementary Fig. 8 Light absorption properties of materials. Tauc plots of transformed Kubelka–Munk function for the prepared samples.

15. Supplementary Fig. 9 In-situ XPS characterization. a XPS Pt 4*f* spectra of TCN@Pt and b the corresponding electron transfer schematics. c XPS Mn 2*p* spectra of HCN@Mn and d the corresponding electron transfer schematics. e XPS Pt 4*f* spectra and f Mn 2*p* spectra of TH1:4@MnPt. g the corresponding electron transfer schematics.

16. Supplementary Fig. 10 TEM characterization of TCN@Pt and HHCN@Mn. a TEM image and **b** HRTEM image of TCN@Pt; **c** TEM image and **d** HRTEM image of HHCN@Mn.

17. Supplementary Fig. 11 XPS characterization of TH1:4@MnPt. a XPS survey

spectra of TH1:4@MnPt. **b** XPS C1*s* spectra of TH1:4@MnPt.

18. Supplementary Fig. 12 Element distribution image of TH1:4@MnPt. a STEM image of TH1:4@MnPt. **b** Total elemental distribution images of TH1:4@MnPt and corresponding elemental distribution images of **c** Pt, **d** Mn, **e** N, **f** O, and **g** C.

19. Supplementary Fig. 13 Photoreduction CO_2 activity and reaction mechanism of TH1:4@PtMn. a Yields of CO and CH_4 of TH1:4@PtMn, and b comparison of CO and CH_4 yields of sample TH1:4@PtMn and TH1:4. c Mechanistic diagram of photocatalytic reduction of CO_2 on TH1:4 and TH:4@PtMn surfaces.

20. Supplementary Fig. 14 Structural model construction. a Side view of bulk HCN. **b** Top view of optimized monolayer HCN with selected crystallographic planes as (002) planes. c Side view of bulk TCN. **d** Top view of optimized monolayer TCN with selected crystallographic planes as (002) planes. **e** Side view and **f** top view of optimized triazine/heptazine crystalline carbon nitride homojunction.

21. Supplementary Fig. 15 Charge density difference of crystallized carbon nitride. a Charge density difference of crystallized carbon nitride homojunction under dark state and **b** illumination, where the isosurface values is 0.003 e/$Å^3$. Yellow represents charge depletion and blue represents charge accumulation. **c** The 2D cross-section view of the charge density difference of a crystallized carbon nitride homojunction under dark and **d** illumination, where the isosurface values are 0.0009 and 0.004 e/$Å^3$. Red represents charge accumulation and blue represents.

22. Supplementary Fig. 16 Performance of CO_2 photoreduction over TCN, HCN, BCN, and TH1:4. a CO and **b** CH_4 yields versus time over the prepared samples.

23. Supplementary Fig. 17 Performance of CO_2 photoreduction over crystallized carbon nitride homojunction. a Yields of CO and **b** CH_4 versus time for homojunction formed by different ratios of TCN and HCN.

24. Supplementary Fig. 18 Structure characterization of crystallized carbon nitride homojunction. a XRD patterns of homojunction formed by different ratios of TCN and HCN. **b** Curves fitted according to the relationship between the ratios of triazine and heptazine phases and the peak areas.

25. Supplementary Fig. 19 In situ XPS characterization of TH1:1@MnPt. XPS survey spectra of TH1:1@MnPt.

26. Supplementary Fig. 20 Dynamically tracking photogenerated electron migration of TH1: 1@MnPt using in situ XPS. a XPS C 1*s* spectra, **b** XPS Mn 2*p* spectra, and **c** XPS Pt 4*f* spectra of TH1: 1@MnPt.

27. Supplementary Fig. 21 Stability evaluation of TH1:4 photocatalytic activity. Cyclic experiment of photoreduction of CO_2 over TH1:4.

28. Supplementary Fig. 22 In situ DRIFT characterization. In situ DRIFT spectra of TH1:4 interacting with H_2O and CO_2 in a dark environment.

29. Supplementary Fig. 23 Exploring the mechanism of photoreduction of CO_2 using in situ DRIFT. a, b In situ DRIFT spectra of TH1:4 interacting with H_2O and CO_2 under illumination, and **c** corresponding 2D contour spectra.

30. Supplementary Fig. 24 O_2 production performance over TH1:4. O_2 produced in the process of photoreduction of CO_2 over TH1:4.

31. Supplementary Fig. 25 Photoelectrochemical performance. a Electrochemical

impedance spectra and **b** transient photocurrent response curves of as-prepared samples.

32. Supplementary Fig. 26 Characterization of redox capacity over prepared samples. LSV curves of the prepared samples.

Supplementary Table. 1 Relationship between vacuum layer thickness and work function of the sample.

Work function of Sample	15 Å	20 Å
TCN	6.03 eV	6.07 eV
HCN	4.69 eV	4.62 eV

Supplementary Table. 2 Energy band structure of crystalline carbon nitride. Band position of the sample.

Sample	Eg (eV)	VB (eV)	CB (eV)
TCN	2.97	1.91	-1.06
HCN	2.80	1.41	-1.39
TH1:4	2.92	1.64	-1.28

Supplementary Table. 3 Detailed information about the structural model. Types and number of atoms in the structural model

Structural Model	Monolayer TCN	Monolayer HCN	Crystallized carbon nitride homojunction
C	6	6	12
N	9	8	17
Li	3	0	3
Cl	1	0	1

Supplementary Table. 4 Composition and ratio of crystal phase in crystallized carbon nitride homojunction. The relationship between the ratio of triazine and heptazine phase and the peak area.

Phase content	Intensity of Triazine	Intensity of Heptszine	Triazine /Heptszine
TH1:8	33811.17	136589.50	0.25
TH1:4	43371.50	139250.16	0.31
TH2:3	45210.50	89522.84	0.51
TH1:1	46700.00	75803.00	0.62
TH3:2	61797.50	79719.67	0.78
TH4:1	69478.50	52609.00	1.32

Supplementary Table. 5 Evaluation of photocatalytic activity. Assessment of CO_2 photoconversion activity over prepared samples.

Sample	Evolution rate (μmol g^{-1} h^{-1})		*Re* (μmol g^{-1} h^{-1})
	CO	CH_4	
BCN	9.28	0.56	23.04
TCN	9.40	1.20	28.4
HCN	16.54	3.64	62.2
TH1:8	15.7	1.00	39.4
TH1:4	19.38	5.29	81.08
TH2:3	16.17	1.46	44.02
TH1:1	17.59	1.50	47.18
TH3:2	12.88	1.04	34.08
TH4:1	12.05	1.2	33.7

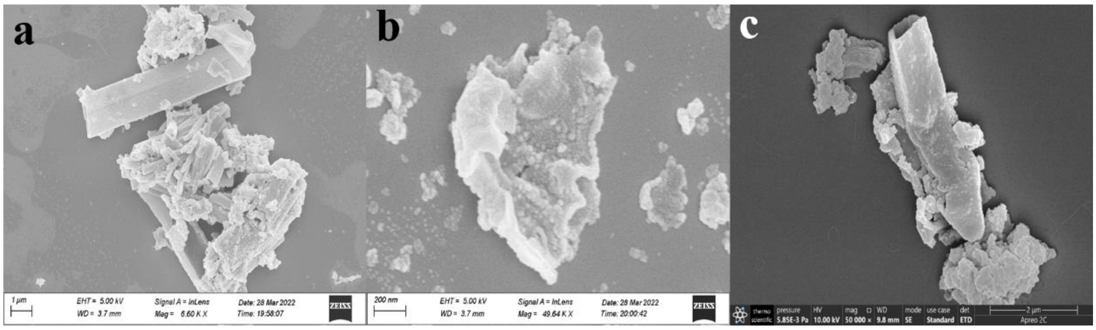

Supplementary Fig. 1 Morphological characterization of the prepared sample. a SEM images of TCN, **b** HCN, and **c** TH1:4.

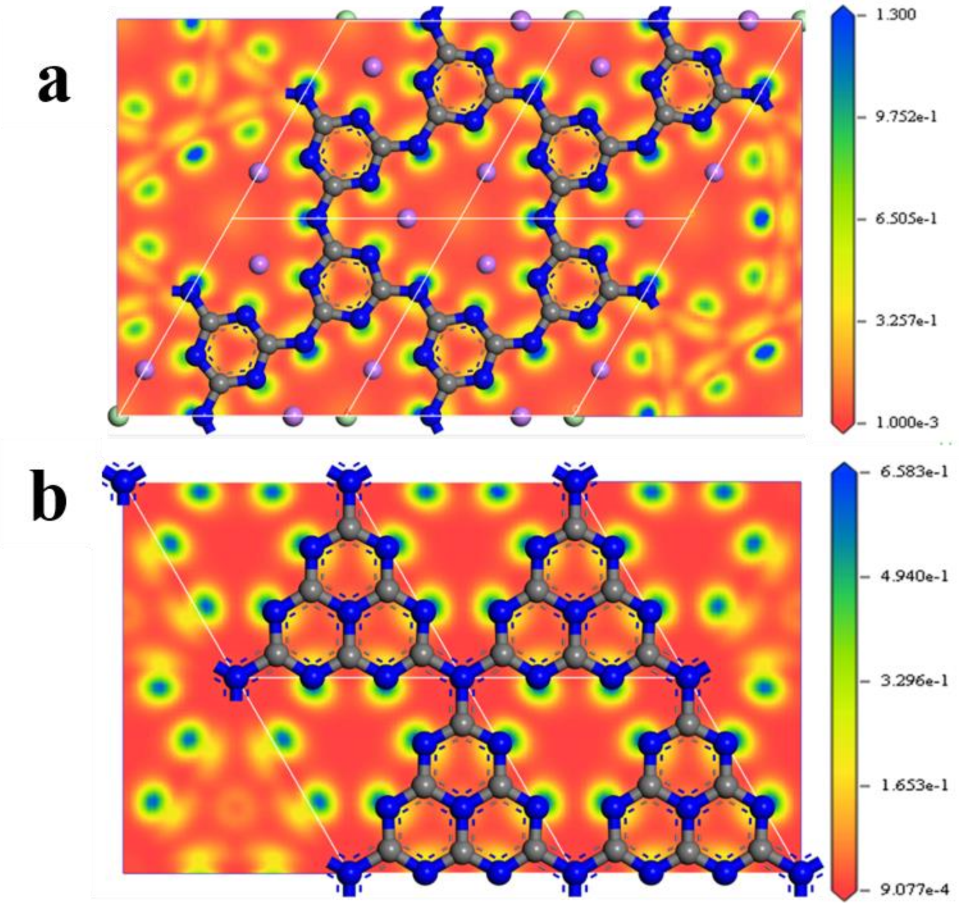

Supplementary Fig. 2 Simulated STM images. a Simulated STM images for TCN and **b** HCN along with the corresponding crystal structures.

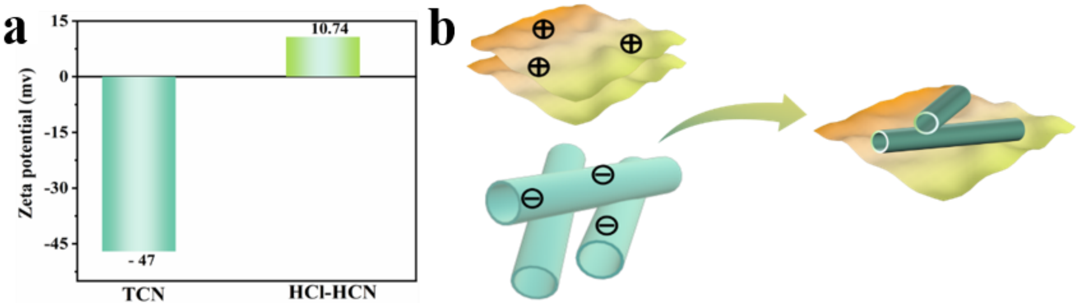

Supplementary Fig. 3 Electrostatic self-Assembly synthesis of TH1:4. a Zeta potential of the as-prepared samples. **b** Illustration of the synthesis process of TH1:4. After hydrochloric acid treatment, the positively charged HCl-HCN and the negatively charged TCN were assembled by electrostatic self-assembly into homojunction.

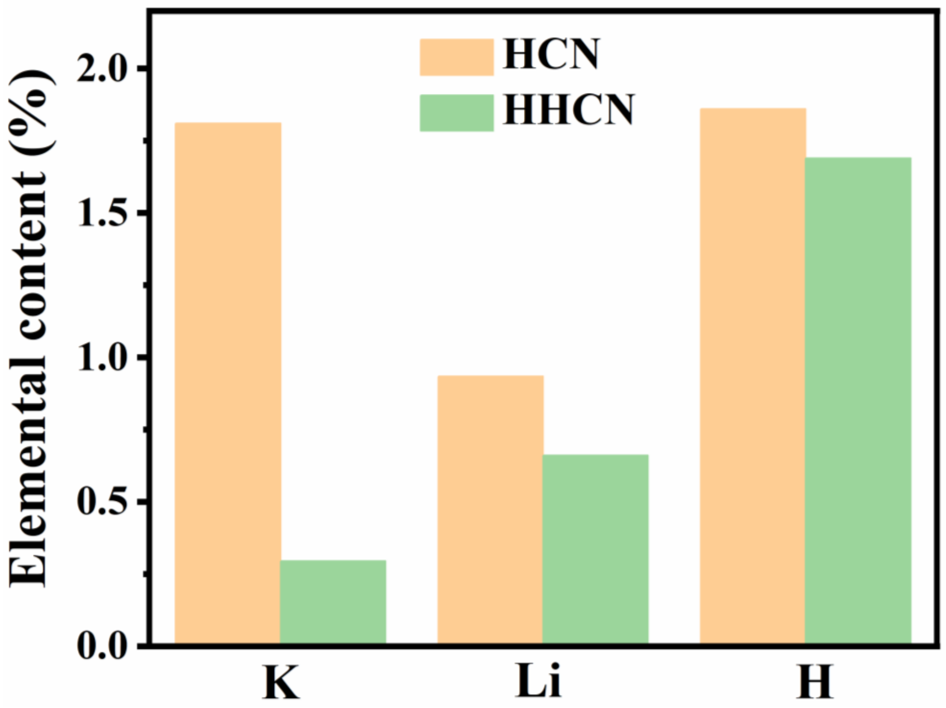

Supplementary Fig. 4 Characterization of elemental content of HCN samples before and after acid treatment. Comparison of elemental content in HCN samples (before acid washing) and HHCN (after acid washing) samples.

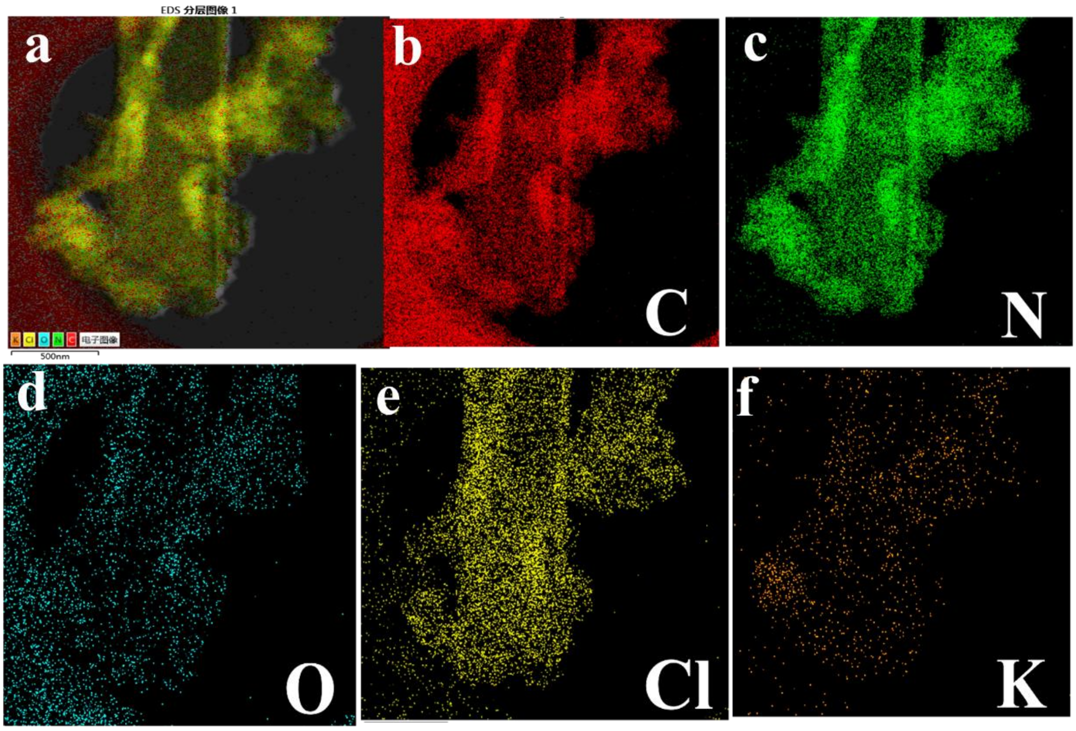

Supplementary Fig. 5 Element distribution image of TH1:4. a The total element distribution image of TH1:4. Elemental mapping images for **b** C, **c** N, **d** O, **e** Cl, and **f** K.

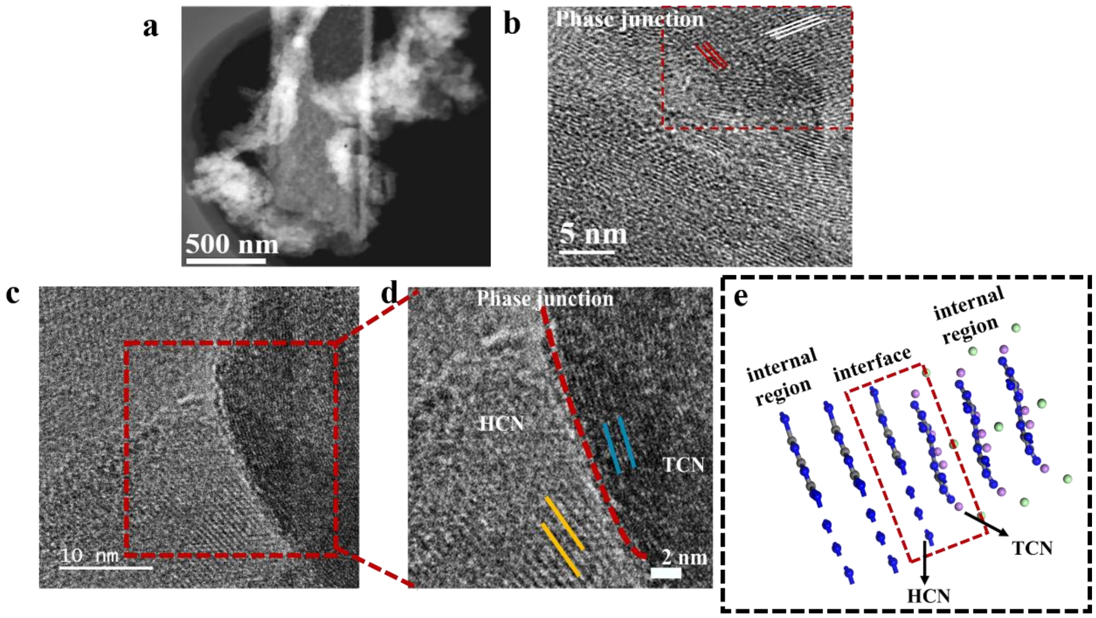

Supplementary Fig. 6 STEM and HRTEM characterization ofTH1:4. a STEM images of TH1:4. **b, c and d** HRTEM images of TH1:4. **e** Schematic diagram of atomic arrangement at interface.

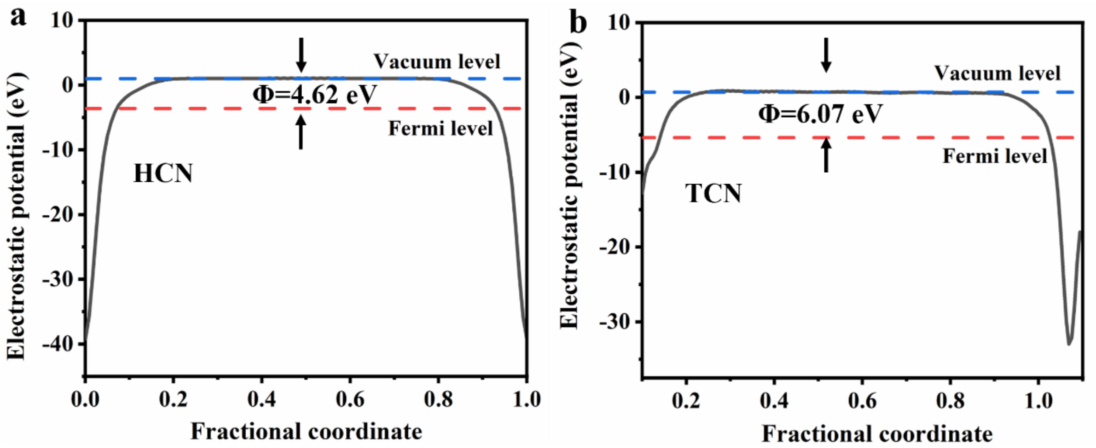

Supplementary Fig. 7 Differences in work functions due to different thicknesses of vacuum layers. a Work function of (002) crystal plane for HCN and b (002) crystal plane for TCN.

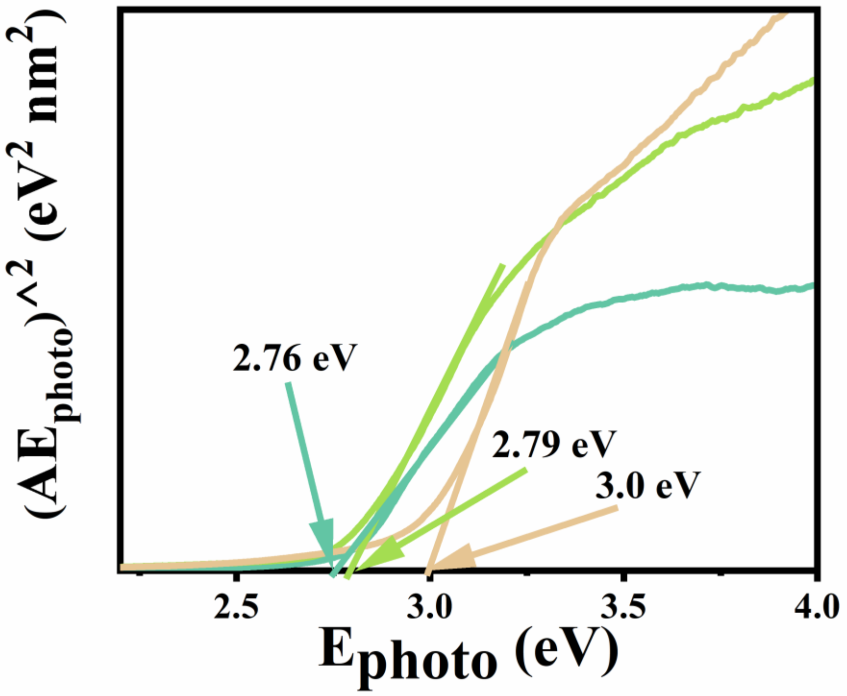

Supplementary Fig. 8 Light absorption properties of materials. Tauc plots of transformed Kubelka–Munk function for the prepared samples.

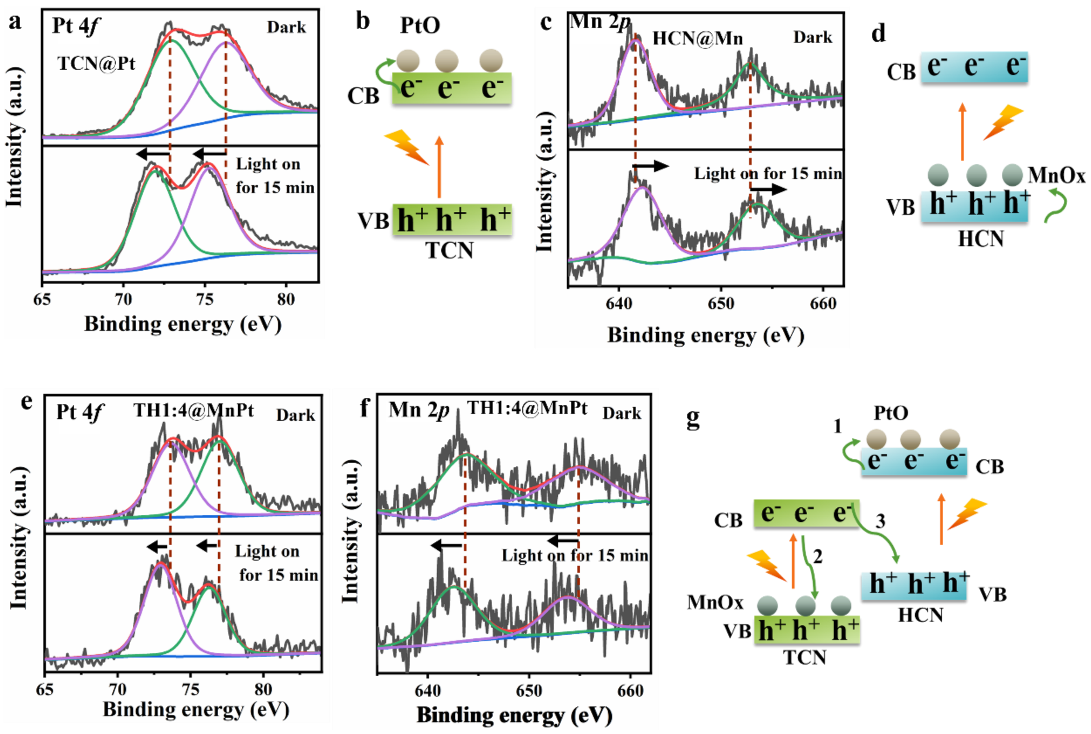

Supplementary Fig. 9 In-situ XPS characterization. a XPS Pt 4*f* spectra of TCN@Pt and b the corresponding electron transfer schematics. c XPS Mn 2*p* spectra of HCN@Mn and d the corresponding electron transfer schematics. e XPS Pt 4*f* spectra and f Mn 2*p* spectra of TH1:4@MnPt. g the corresponding electron transfer schematics.

The electron transfer path of the TCN@Pt sample under photoexcitation conditions is depicted in Supplementary Fig. 9a. The spectra of Pt 4*f* is shifted to the left after illumination compared to that of the dark state, which means that the photoelectrons excited by light on TCN are transferred to Pt species (Supplementary Fig. 9b). For HCN@Mn, the spectrum of Mn 2*p* is shifted to the right after illumination compared with that of the dark state (Supplementary Fig. 9c), which means that Mn loses electrons under photoexcitation conditions and electrons may be transferred from Mn to HCN. In other words, holes in the valence band of HCN are transferred to Mn for further oxidation of MnOx (Supplementary Fig. 9d). Fig. 9e and 9f depict the electron migration paths of TH1:4@MnPt under photoexcitation conditions. Th1:4@MnPt means that Pt is loaded on the conduction band of HCN and Mn is loaded on the valence band of TCN. The binding energy of Pt 4*f* becomes smaller in TH1:4@MnPt upon photoexcitation compared to that of the dark state, which indicates that electrons are

transferred from HCN to Pt and this process dominates (Supplementary Fig. 9g). The binding energy of Mn 2*p* in TH1:4@MnPt also appears to decrease upon photoexcitation compared to that of the dark state, which indicates the transfer of electrons from the conduction band of TCN to Mn and somehow mitigates the carrier recombination due to the role of Mn in receiving electrons.

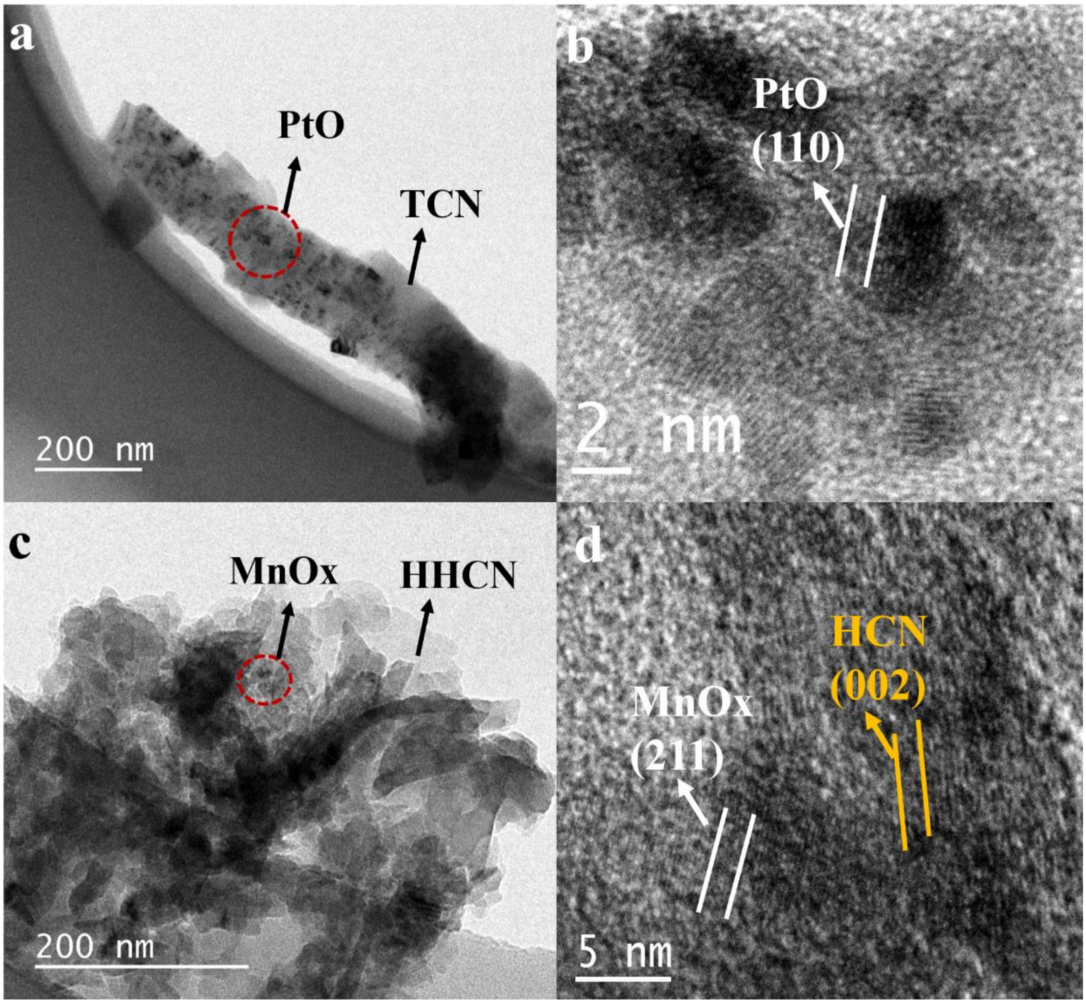

Supplementary Fig. 10 TEM characterization of TCN@Pt and HHCN@Mn. a TEM image and **b** HRTEM image of TCN@Pt; **c** TEM image and **d** HRTEM image of HHCN@Mn.

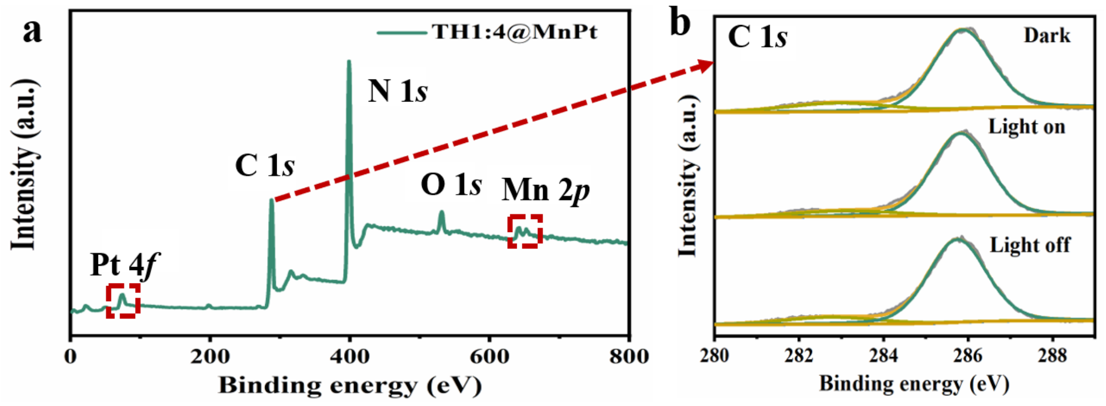

Supplementary Fig. 11 XPS characterization of TH1:4@MnPt. a XPS survey spectra of TH1:4@MnPt. **b** XPS C1*s* spectra of TH1:4@MnPt.

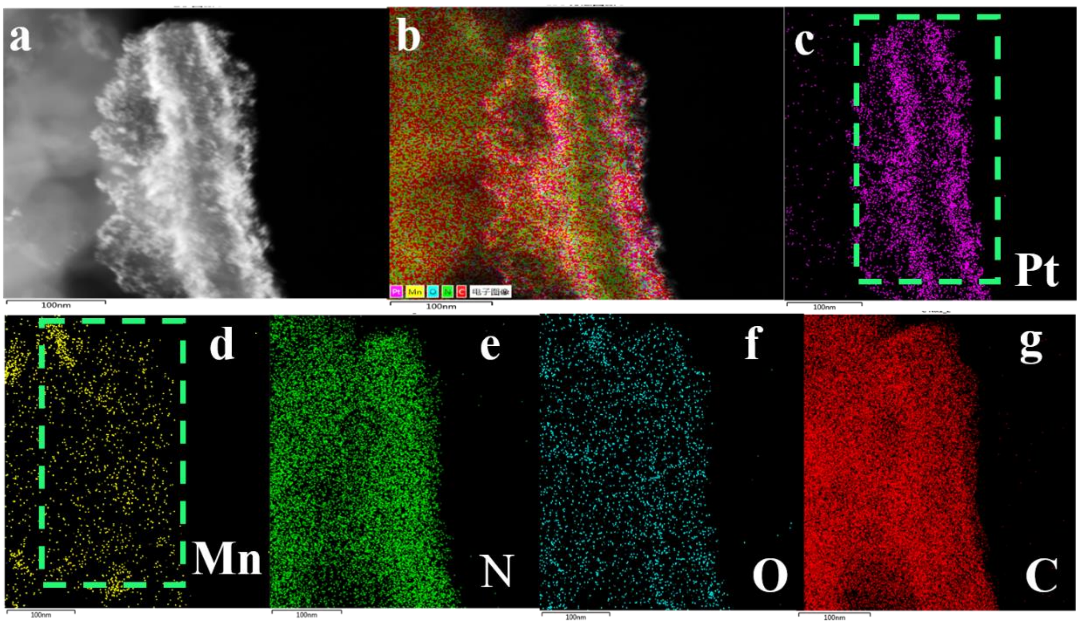

Supplementary Fig. 12 Element distribution image of TH1:4@MnPt. a STEM image of TH1:4@MnPt. **b** Total elemental distribution images of TH1:4@MnPt and corresponding elemental distribution images of **c** Pt, **d** Mn, **e** N, **f** O, and **g** C.

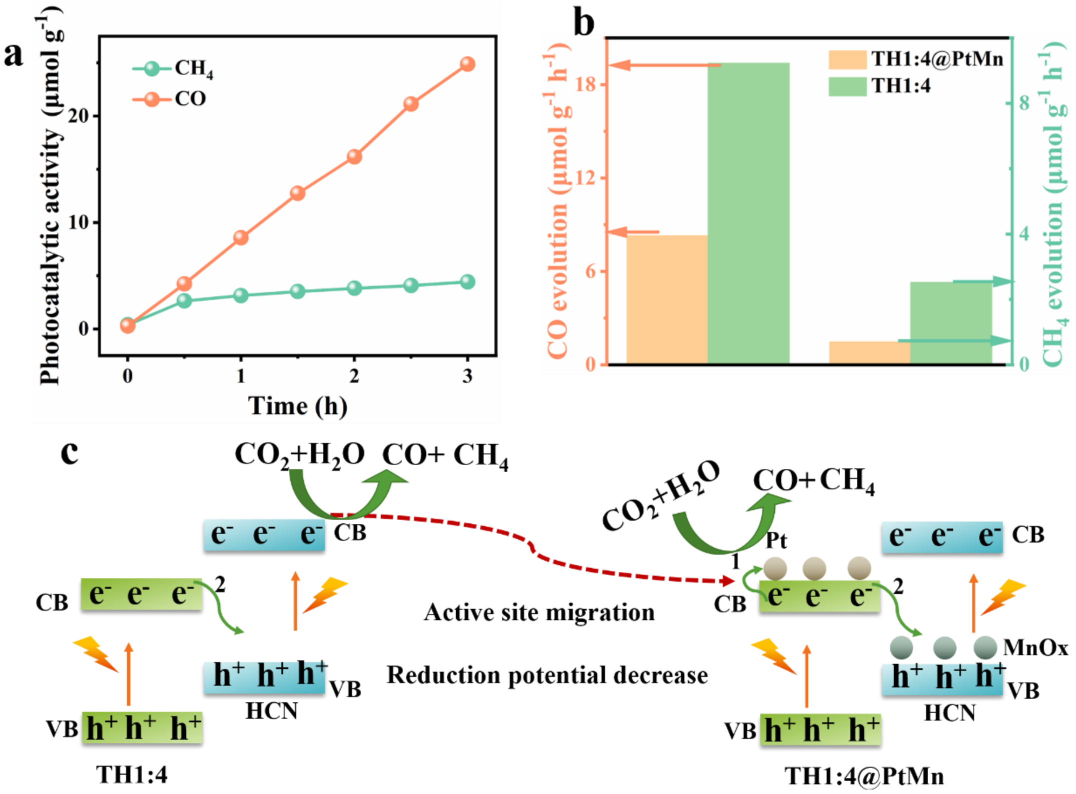

Supplementary Fig. 13 Photoreduction CO_2 activity and reaction mechanism of TH1:4@PtMn. a Yields of CO and CH_4 of TH1:4@PtMn, and b comparison of CO and CH_4 yields of sample TH1:4@PtMn and TH1:4. c Mechanistic diagram of photocatalytic reduction of CO_2 on TH1:4 and TH:4@PtMn surfaces.

We investigated the photocatalytic activity of TH1:4@PtMn and found that the photocatalytic activity of TH1:4 decreased significantly after loading the double co-catalyst and exhibited a similar photocatalytic activity to that of TCN (Supplementary Fig.13a and 13b). Combined with the available data, the possible reason is that the loading of Pt species leads to the migration of the active site from the conduction band side of HCN to the conduction band side of TCN, thus losing part of the reduction driving force (Supplementary Fig.13c). The loading of Pt species on TCN is actually detrimental to interfacial electron transfer. As analyzed in the main text, Pt is involved in two electron transfer processes, and the electron transfer from TCN to Pt dominates, with a large number of electrons concentrated on Pt, thus becoming the main site for CO_2 reduction.

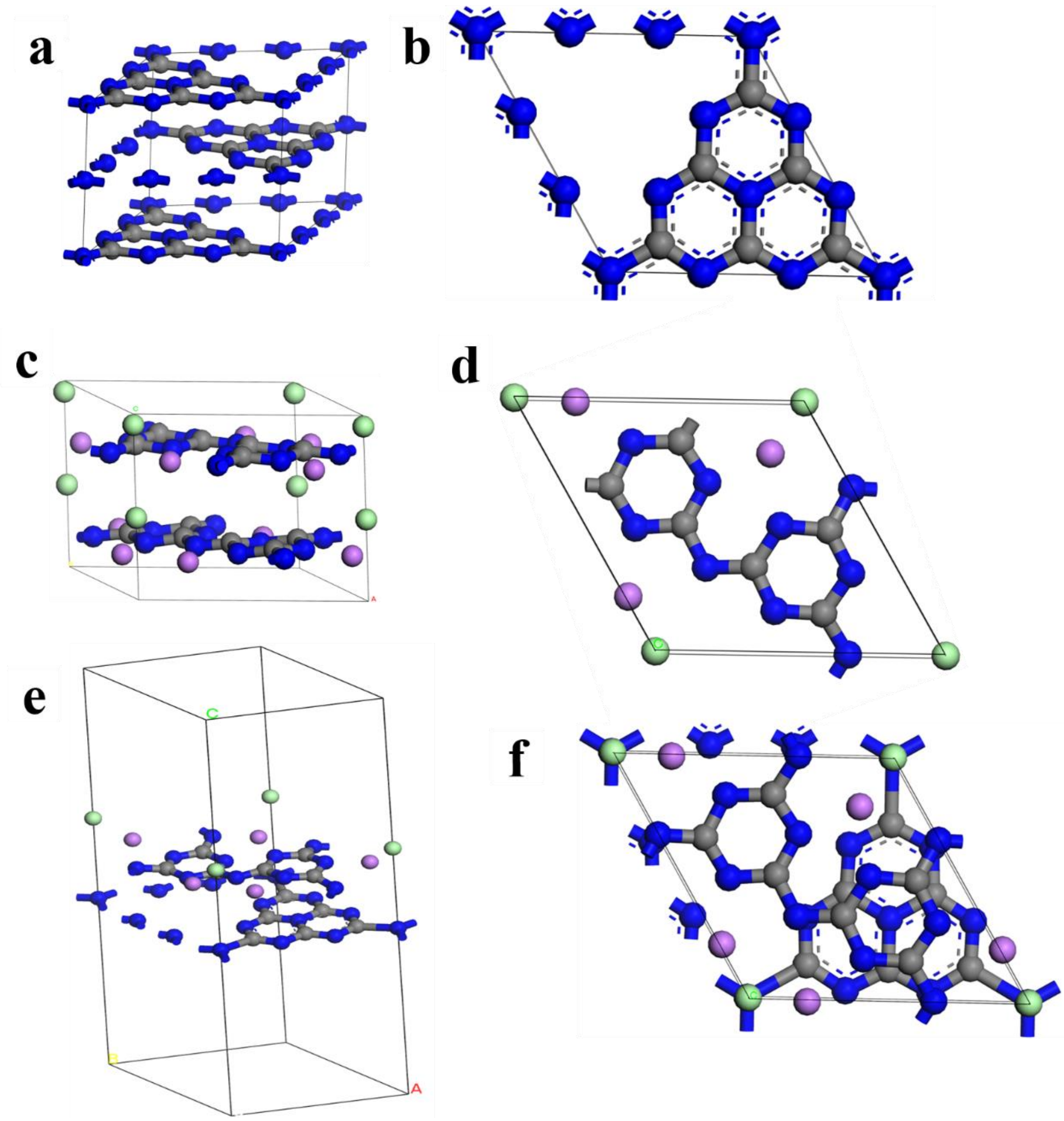

Supplementary Fig. 14 Structural model construction a Side view of bulk HCN. **b** Top view of optimized monolayer HCN with selected crystallographic planes as (002) planes. c Side view of bulk TCN. **d** Top view of optimized monolayer TCN with selected crystallographic planes as (002) planes. **e** Side view and **f** top view of optimized triazine/heptazine crystalline carbon nitride homojunction.

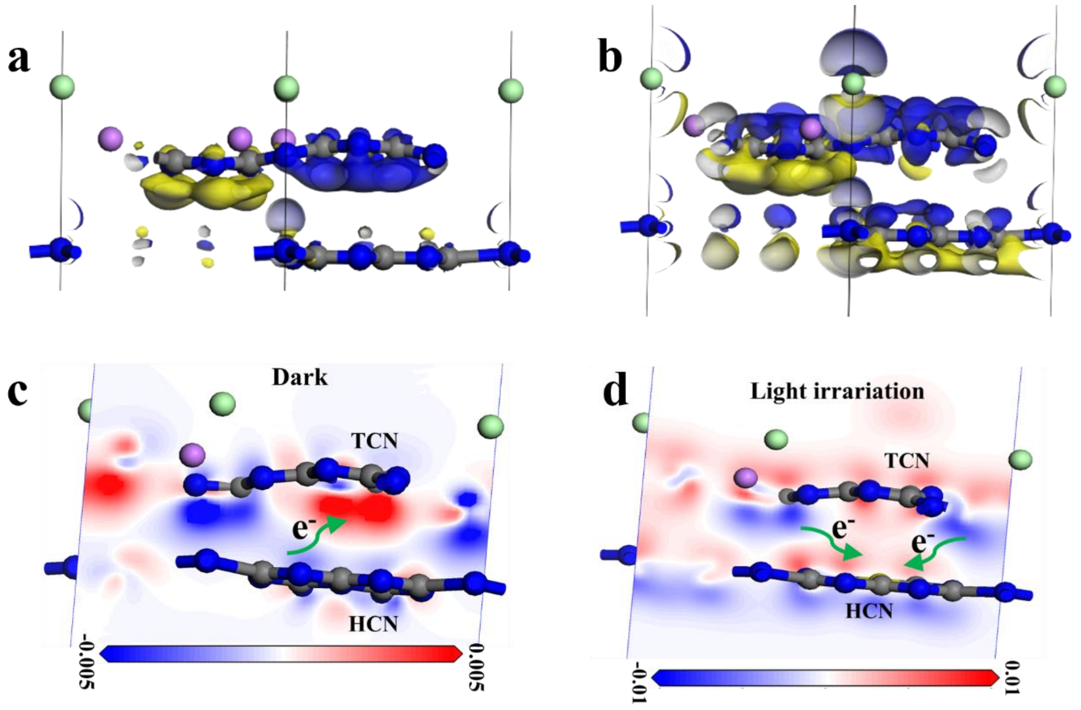

Supplementary Fig. 15 Charge density difference of crystallized carbon nitride. a Charge density difference of crystallized carbon nitride homojunction under dark state and **b** illumination, where the isosurface values is 0.003 e/Å^3. Yellow represents charge depletion and blue represents charge accumulation. **c** The 2D cross-section view of the charge density difference of a crystallized carbon nitride homojunction under dark and **d** illumination, where the isosurface values are 0.0009 and 0.004 e/Å^3. Red represents charge accumulation and blue represents.

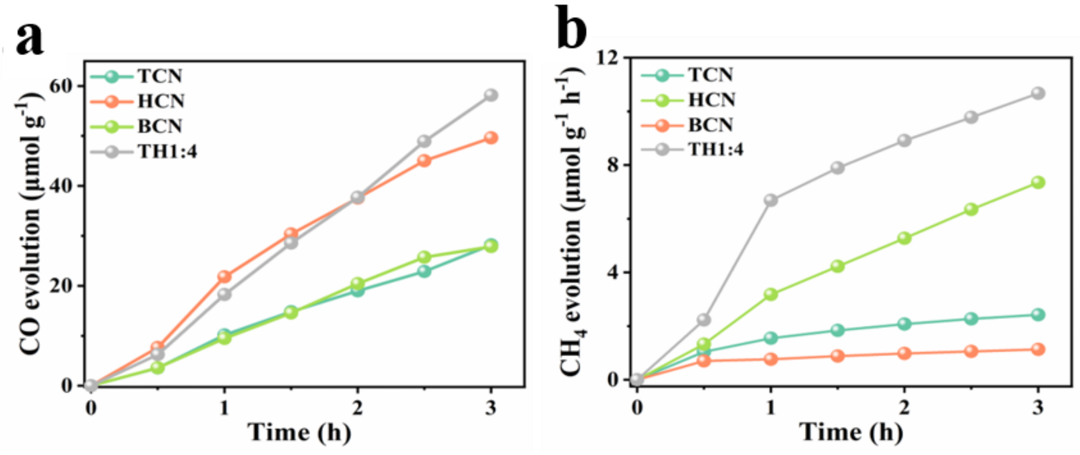

Supplementary Fig. 16 Performance of CO_2 photoreduction over TCN, HCN, BCN, and TH1:4. a CO and **b** CH_4 yields versus time over the prepared samples.

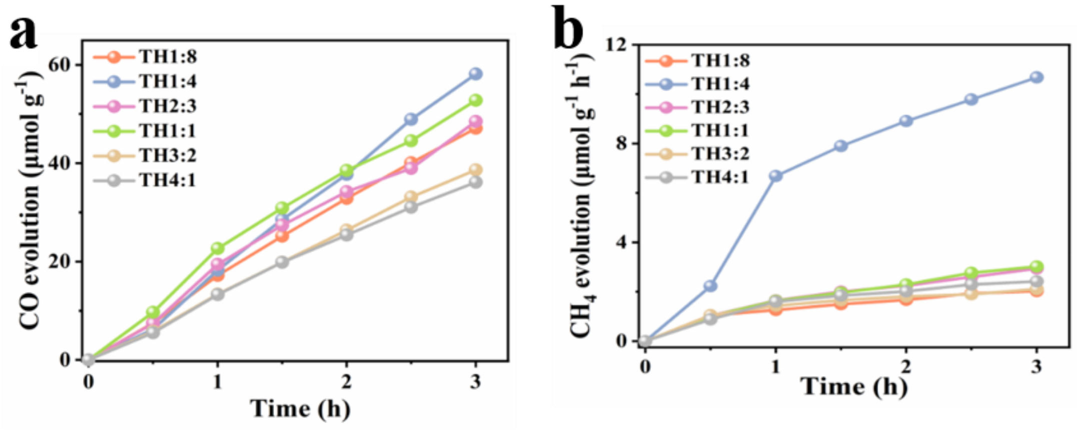

Supplementary Fig. 17 Performance of CO_2 photoreduction over crystallized carbon nitride homojunction. a Yields of CO and **b** CH_4 versus time for homojunction formed by different ratios of TCN and HCN.

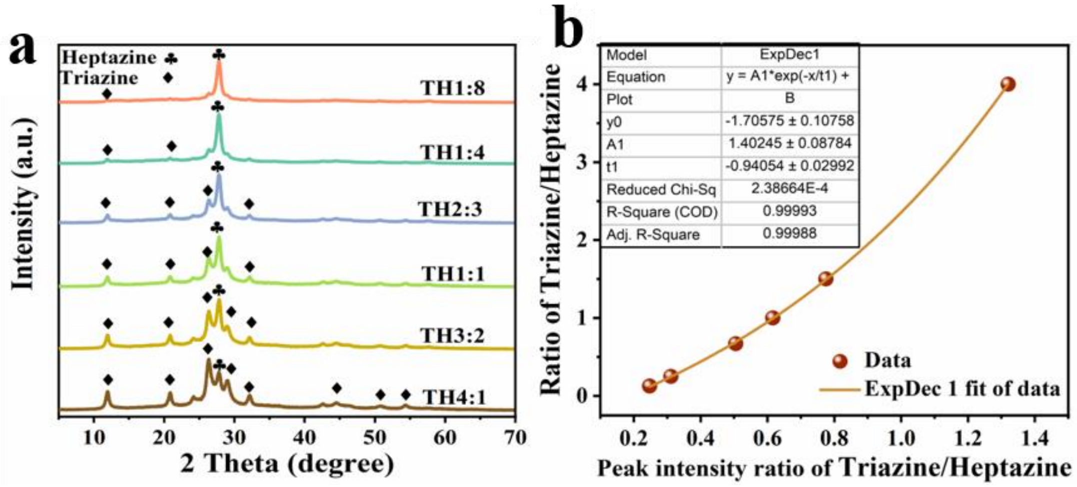

Supplementary Fig. 18 Structure characterization of crystallized carbon nitride homojunction. a XRD patterns of homojunction formed by different ratios of TCN and HCN. **b** Curves fitted according to the relationship between the ratios of triazine and heptazine phases and the peak areas.

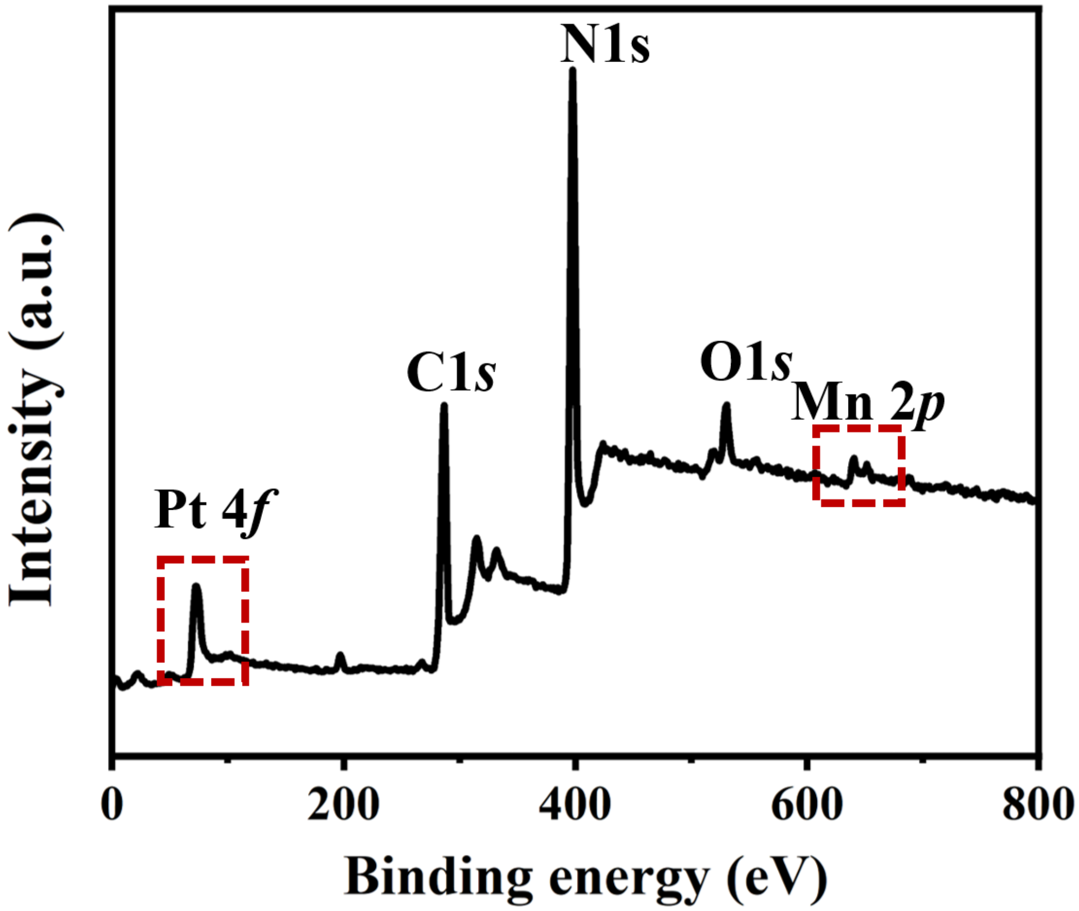

Supplementary Fig. 19 In situ XPS characterization of TH1:1@MnPt. XPS survey spectra of TH1:1@MnPt.

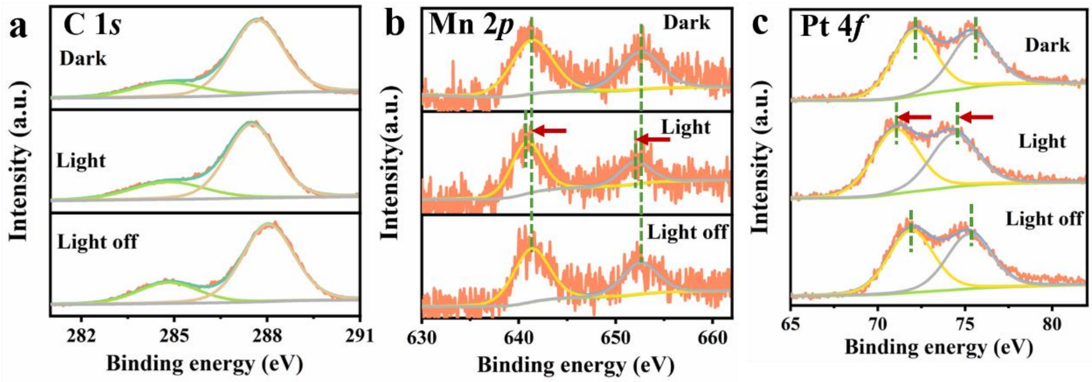

Supplementary Fig. 20 Dynamically tracking photogenerated electron migration of TH1: 1@MnPt using in situ XPS. **a** XPS C 1*s* spectra, **b** XPS Mn 2*p* spectra, and **c** XPS Pt 4*f* spectra of TH1: 1@MnPt.

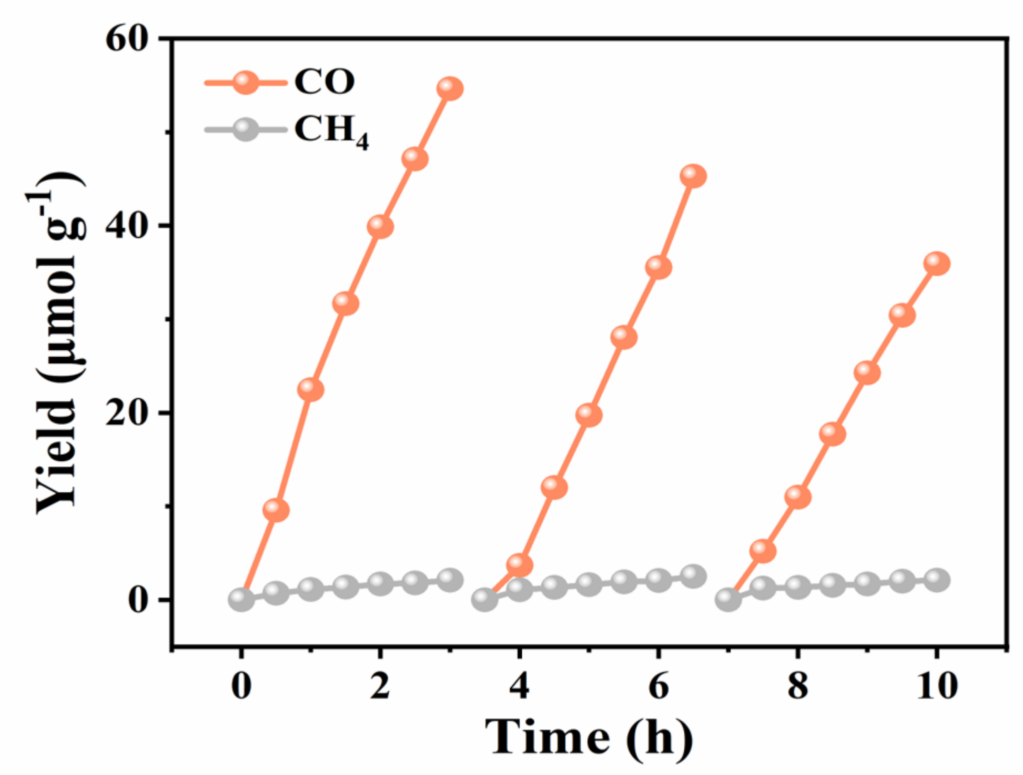

Supplementary Fig. 21 Stability evaluation of TH1:4 photocatalytic activity. Cyclic experiment of photoreduction of CO_2 over TH1:4.

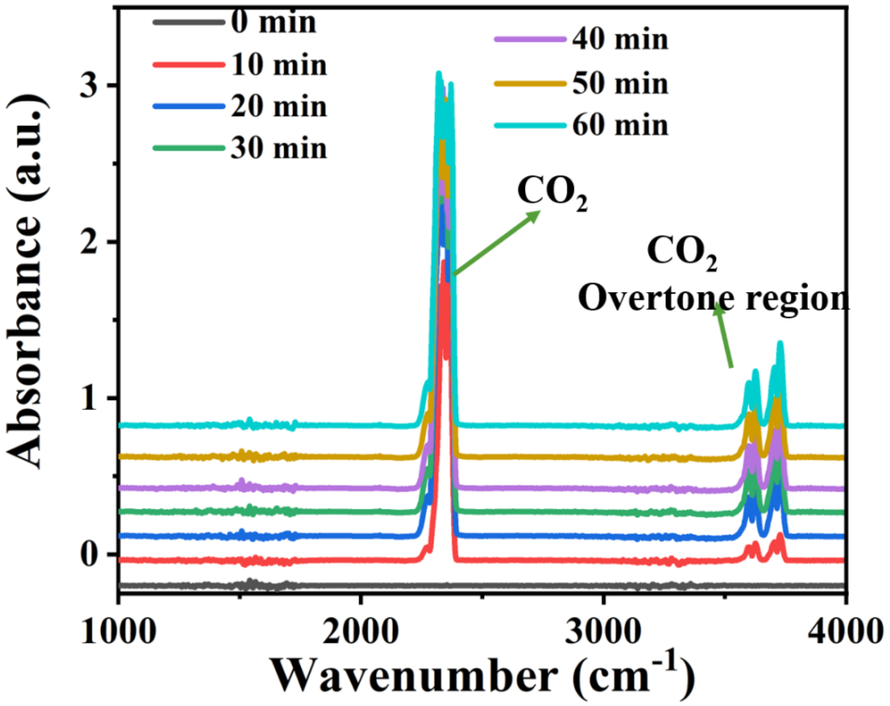

Supplementary Fig. 22 In situ DRIFT characterization. In situ DRIFT spectra of TH1:4 interacting with H_2O and CO_2 in a dark environment.

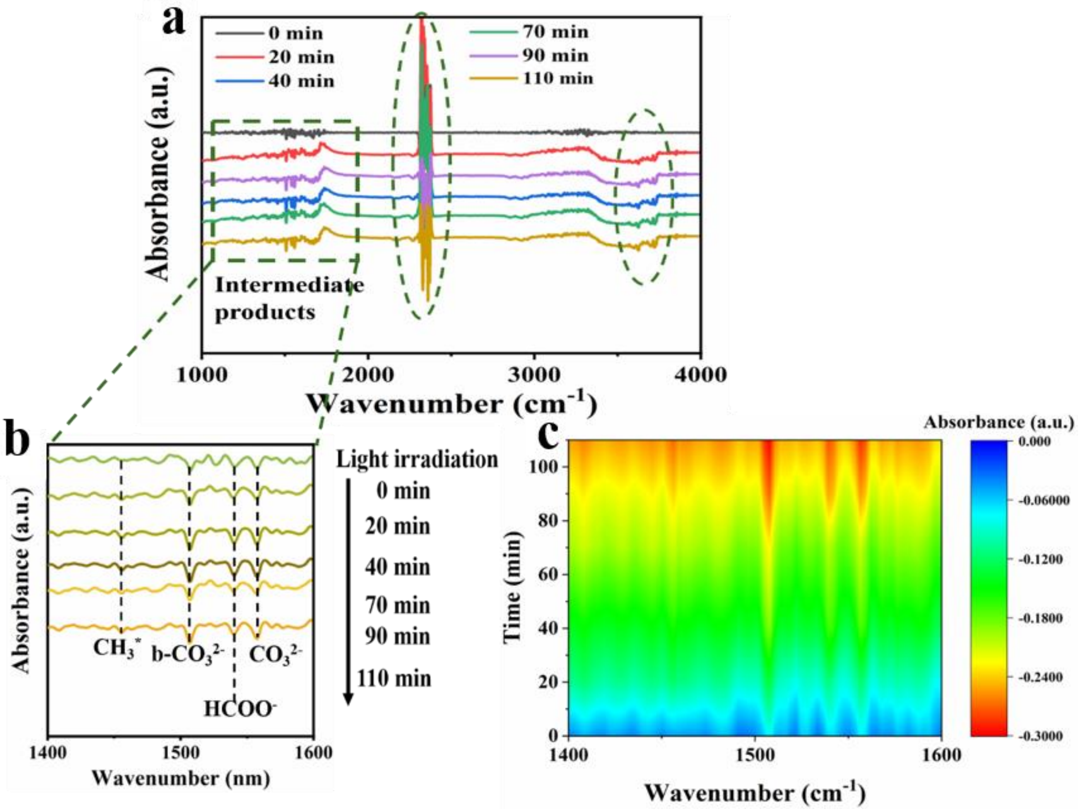

Supplementary Fig. 23 Exploring the mechanism of photoreduction of CO_2 using in situ DRIFT. a,b In situ DRIFT spectra of TH1:4 interacting with H_2O and CO_2 under illumination, and **c** corresponding 2D contour spectra.

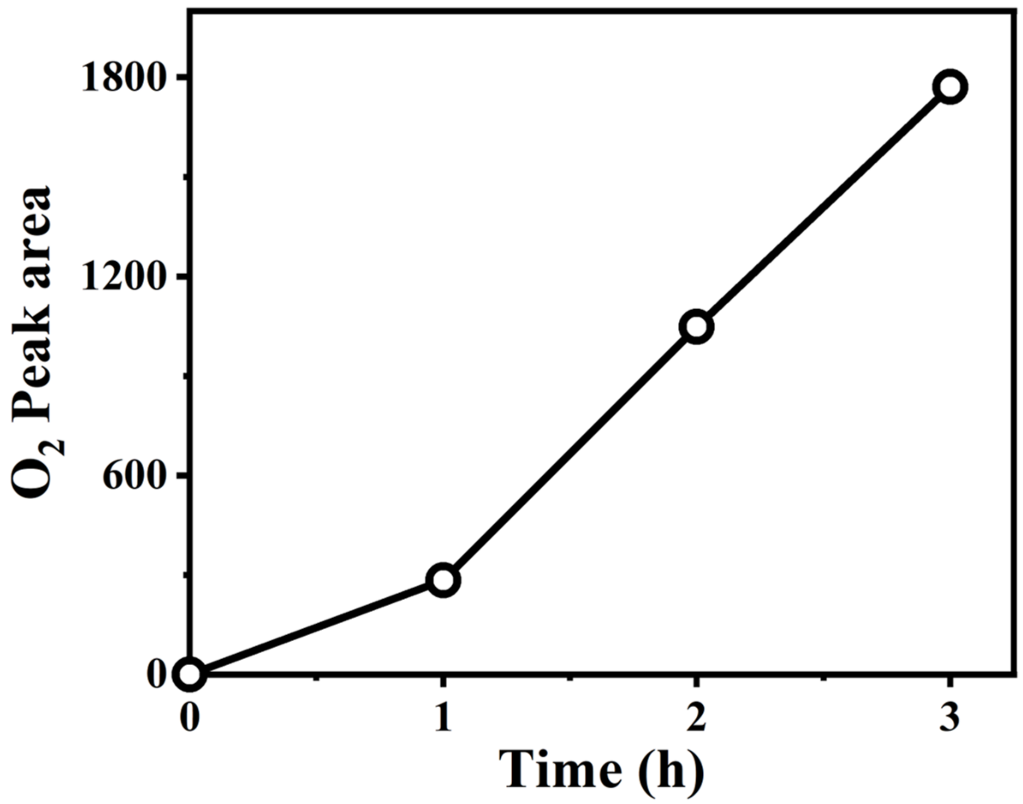

Supplementary Fig. 24 O_2 production performance over TH1:4. O_2 produced in the process of photoreduction of CO_2 over TH1:4.

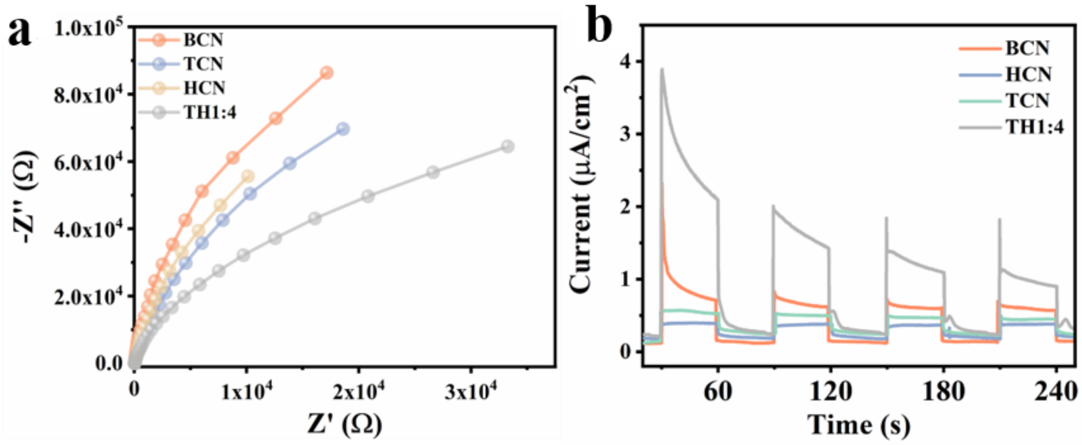

Supplementary Fig. 25 Photoelectrochemical performance. a Electrochemical impedance spectra and **b** transient photocurrent response curves of as-prepared samples.

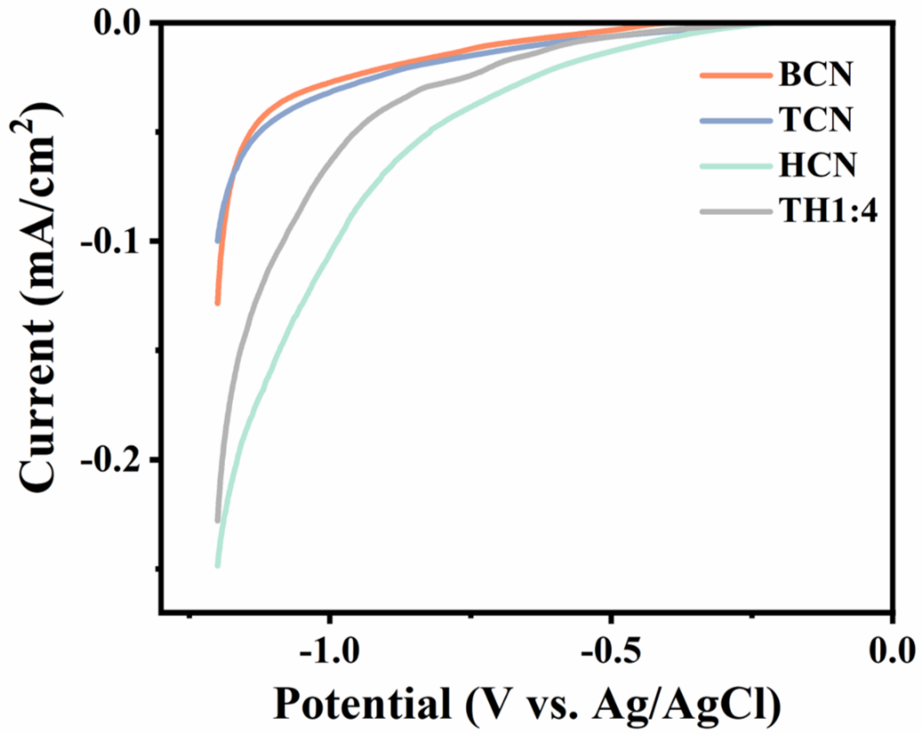

Supplementary Fig. 26 Characterization of redox capacity over prepared samples.

LSV curves of the prepared samples.
